# Prehospital stroke mimics in the Stockholm Stroke Triage System

**DOI:** 10.3389/fneur.2022.939618

**Published:** 2022-08-18

**Authors:** Mimmi Sjöö, Annika Berglund, Christina Sjöstrand, Einar E. Eriksson, Michael V. Mazya

**Affiliations:** ^1^Department of Clinical Neuroscience, Karolinska Institutet, Stockholm, Sweden; ^2^Department of Neurology, Karolinska University Hospital, Stockholm, Sweden; ^3^Department of Neurology, Danderyd Hospital, Stockholm, Sweden

**Keywords:** acute ischemic stroke, stroke mimics, prehospital triage, telemedicine, thrombectomy

## Abstract

**Introduction:**

In 2017, Stockholm implemented a new prehospital stroke triage system (SSTS) directing patients with a likely indication for thrombectomy to the regional comprehensive stroke center (CSC) based on symptom severity and teleconsultation with a physician. In Stockholm, 44% of patients with prehospital code stroke have stroke mimics. Inadvertent triage of stroke mimics to the CSC could lead to inappropriate resource utilization.

**Aims:**

To compare the characteristics between (1) triage-positive stroke mimics and stroke (TP mimics and TP stroke) and (2) triage-negative stroke mimics and stroke (TN mimics and TN stroke) and to (3) compare the distribution of stroke mimic diagnoses between triage-positive and triage-negative cases.

**Methods:**

This prospective observational study collected data from October 2017 to October 2018, including 2,905 patients with suspected stroke who were transported by code-stroke ambulance to a Stockholm regional hospital. Patients directed to the CSC were defined as triage-positive. Those directed to the nearest stroke center were defined as triage-negative.

**Results:**

Compared to individuals with TP stroke (*n* = 268), those with TP mimics (*n* = 55, median 64 vs. 75 years, *P* < 0.001) were younger and had lower NIHSS score (median 7 vs. 15, *P* < 0.001). Similarly, those with TN mimics (*n* = 1,221) were younger than those with TN stroke (n = 1,361, median 73 vs. 78 years, *P* < 0.001) and had lower NIHSS scores (median 2 vs. 4, *P* < 0.001). Functional paresis was more common in those with TP mimics than in those with TN mimics, 18/55 (32.7%) vs. 82/1,221 (6.7%), *P* < 0.001. Systemic infection was less common in those with TP mimics than in those with TN mimics, 1/55 (1.8%) vs. 160/1,221 (13.1%), *P* < 0.011. There was a trend toward “syncope, hypotension, or other cardiovascular diagnosis” being less common in those with TP mimics than in those with TN mimics, 1/55 (1.8%) vs. 118/1,221 (9.7%), *P* < 0.055.

**Conclusions:**

In the SSTS, those with triage-positive and triage-negative stroke mimics were younger and had less severe symptoms than patients with stroke. All patients with TP mimics who had hemiparesis but overall exhibited less severe symptoms against true stroke but more severe symptoms than those with TN mimics were triaged to the nearest hospital. Over-triage of functional paresis to the CSC was relatively common. Meanwhile, a large majority of cases with minor symptoms caused by stroke mimics was triaged correctly by the SSTS to the nearest stroke center.

## Introduction

In acute ischemic stroke (AIS), treatment with endovascular thrombectomy (EVT) and intravenous thrombolysis (IVT) has the greatest effect when initiated within the first h to several h after onset (1, 2). EVT availability is limited to comprehensive or thrombectomy-ready stroke centers (CSCs or TSCs), while IVT can be administered in all stroke centers, including primary stroke centers (PSCs). Interhospital transfer from PSCs to CSCs delays treatment and is associated with worse outcomes (3). However, mass bypass of PSCs could overwhelm CSC capacity with EVT-ineligible cases. This highlights the need for an accurate prehospital triage system.

In the prehospital setting, clinical algorithms are used to detect signs of stroke and large artery occlusion (LAO). However, diagnosing AIS in the prehospital setting is challenging, and frequently, other conditions, so called stroke mimics, are mistaken for stroke.

Prior to the Stockholm Stroke Triage Project, prehospital guidelines mandated triage using a Swedish, modified, version of the Face-Arm-Speech-Time (FAST) test, allowing for both arm or leg weakness (4). Patients with suspected stroke were transported with first-priority ambulance to the nearest hospital for initial assessment and treatment. Patients potentially eligible for thrombectomy were subsequently transported to the CSC in the Stockholm region. A new workflow, the Stockholm Stroke Triage System (SSTS), was implemented in October 2017. This combines the assessment of hemiparesis with ambulance-hospital teleconsultation to direct patients with high likelihood of LAO and EVT indication directly to the CSC, bypassing PSCs.

The SSTS has a high accuracy for predicting LAO and indication for EVT (5, 6). The implementation of SSTS has resulted in a 69-min reduction of symptom onset to arterial puncture time in EVT and improved patient outcomes (7). However, 44% of all suspected stroke cases managed in the SSTS are ultimately diagnosed with a stroke mimic (5).

To assess if resource utilization could be optimized and care pathways for patients with stroke mimic in the SSTS could be improved, we wanted to identify which types of stroke mimics tend to be routed to the CSC. By assessing differences in prehospital characteristics between stroke mimics and true stroke triaged to the CSC and the nearest PSC, we hoped to find any patterns helpful for improvement of the teleconsultation. Improving mimic triage is not only important for patients with stroke mimic but could also indirectly benefit those with true stroke. Over-triage could lead to increased patient volume routed to CSC, which could lead to resource conflicts and delay in treatment if the capacity to manage parallel cases is exhausted (8).

We aimed to compare characteristics between (1) triage-positive stroke mimics and stroke (TP mimics and TP stroke) and (2) triage-negative stroke mimics and stroke (TN mimics and TN stroke), and (3) compare the distribution of stroke mimic diagnoses between triage-positive and triage-negative cases.

## Materials and methods

This was an observational cohort study using prospectively collected data between 10 October 2017 and 9 October 2018. The inclusion criteria were prehospital suspicion of stroke and transport with code-stroke ground ambulance to a hospital in the Stockholm region, and patients aged 18 years or above. The exclusion criteria were transport to a hospital by means other than a priority one ambulance or transport from outside the Stockholm region and stroke during in-hospital care for another condition.

The Swedish Ethical Review Authority (ERA) gave approval for the project. Need for active consent was waved. Patients or family were informed in writing of the right to opt out of study data collection.

**Figure 1:**
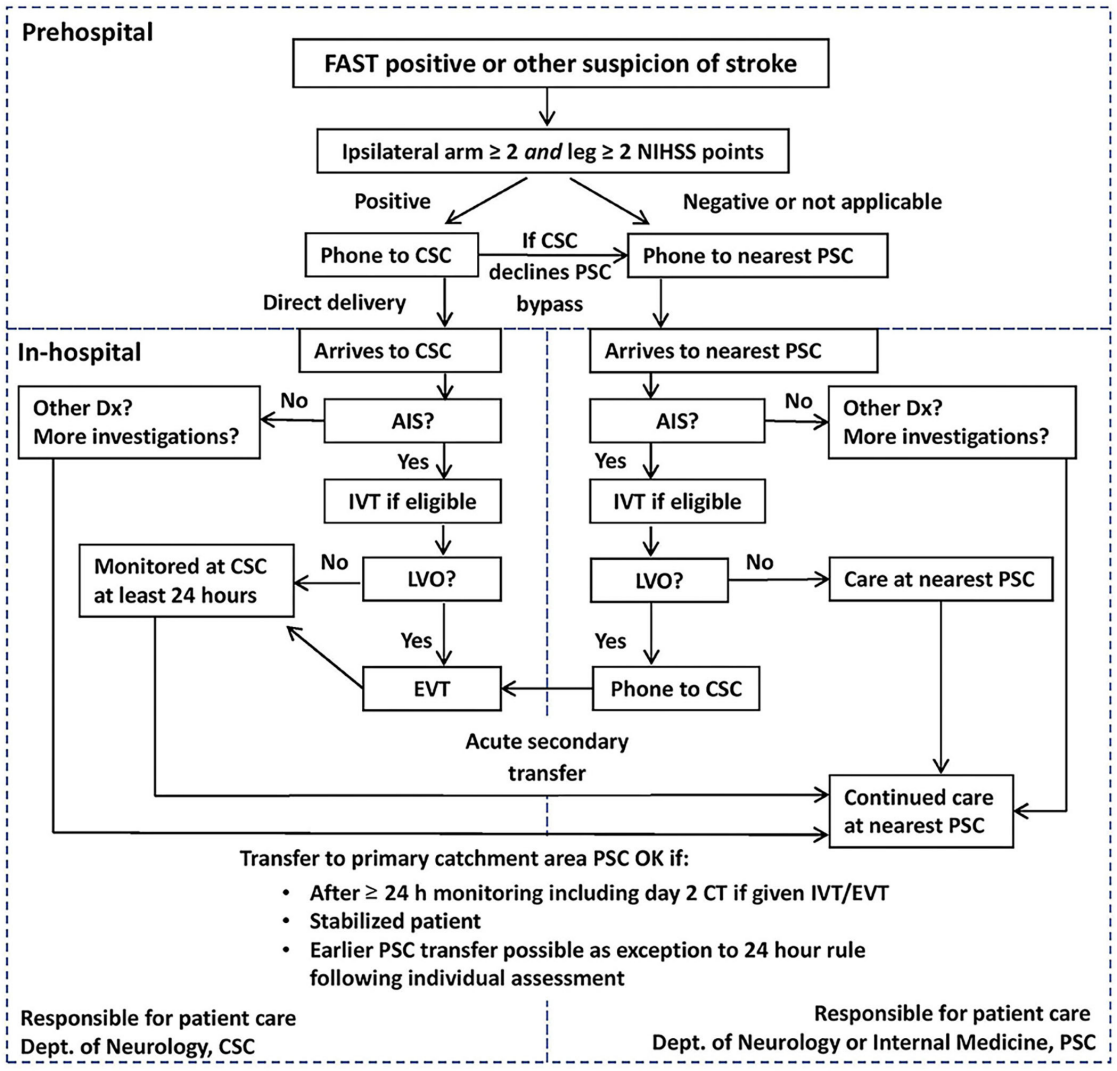
Flowchart of the Stockholm Stroke Triage System.

The SSTS was implemented on 10 October 2017 in the Stockholm region (population 2.3 M, area 6,519 km^2^), which is served by six primary stroke centers (PSCs) and one comprehensive stroke center (CSC). The triage process starts with an ambulance nurse suspecting stroke as a potential cause of symptoms either *via* the FAST test or for any other clinical reason. The next step is to assess for hemiparesis using the motor items of the NIH Stroke Scale (NIHSS), where ≥2 points in each of the affected extremities are considered positive. This test is named the A2L2 test (arm two or more, leg two or more). If the patient cannot keep an arm and ipsilateral leg raised for 10 and 5 s, respectively, the A2L2 test is positive. The test is not applicable to patients with seizures, unconsciousness, or bilateral paresis. The final step is teleconsultation between the ambulance nurse and a stroke neurologist at the CSC in A2L2-positive cases or a pre-notification to the nearest PSC in A2L2-negative or inapplicable cases. A flowchart of the triage system is shown in Figure 1.

In the Stockholm region, all ambulances are operated by a crew of two, with at least one specialist ambulance nurse with 3 years of university education and 1 year of specialist training in either prehospital care or anesthesia. Prior to the implementation of the SSTS, ambulance nurses were educated by web-based training and live lectures on the SSTS and stroke scales used.

Data were collected on baseline demographics, imaging and clinical parameters, treatments, time measures, and final diagnosis. Data sources were the region-wide electronic health record systems for emergency health services and for in-hospital, outpatient, and primary care services along with the regional radiological picture archiving and communication system.

Triage-positive (TP) cases were defined as patients with a positive A2L2 test and those who were, after teleconsultation, triaged to the CSC with suspected LAO and no EVT contraindications. Triage-negative (TN) cases were defined as patients with a negative A2L2 test or patients with a positive A2L2 test who, after teleconsultation, were directed to a PSC because of EVT contraindications. In cases where the closest hospital was the CSC, patients were still triaged the same way, by the A2L2 test and teleconsultation. If the patient was triage-negative they were still taken to the CSC, as it was the nearest hospital. Meanwhile, for the purposes of the study, they were still classified as triage negative. Contraindications for EVT were: >24 h since time of last known well, severe co-morbidity with a pre-stroke life expectancy less than 3 months, and pre-stroke mRS of 4–5.

Stroke mimics were defined as a main discharge diagnosis other than stroke or transient ischemic attack (TIA). These were classified into standardized categories: epileptic seizure, functional neurological disorder, primary headache disorder, etc. The final diagnosis of stroke was made on discharge by synthesis of clinical and radiological findings including CT or MR as deemed clinically appropriate on a case-by-case basis.

Continuous and ordinal variables were reported as medians with the interquartile range (IQR). Statistical significance of differences was assessed by the Mann-Whitney U test. Statistical significance of differences between proportions was assessed by the Pearson chi-square test or Fisher's exact test as appropriate. A difference with a *p*-value <0.05 was considered statistically significant. SPSS version 27 (IBM, Armonk, NY, United States) was used for data analysis.

## Results

**Figure 2:**
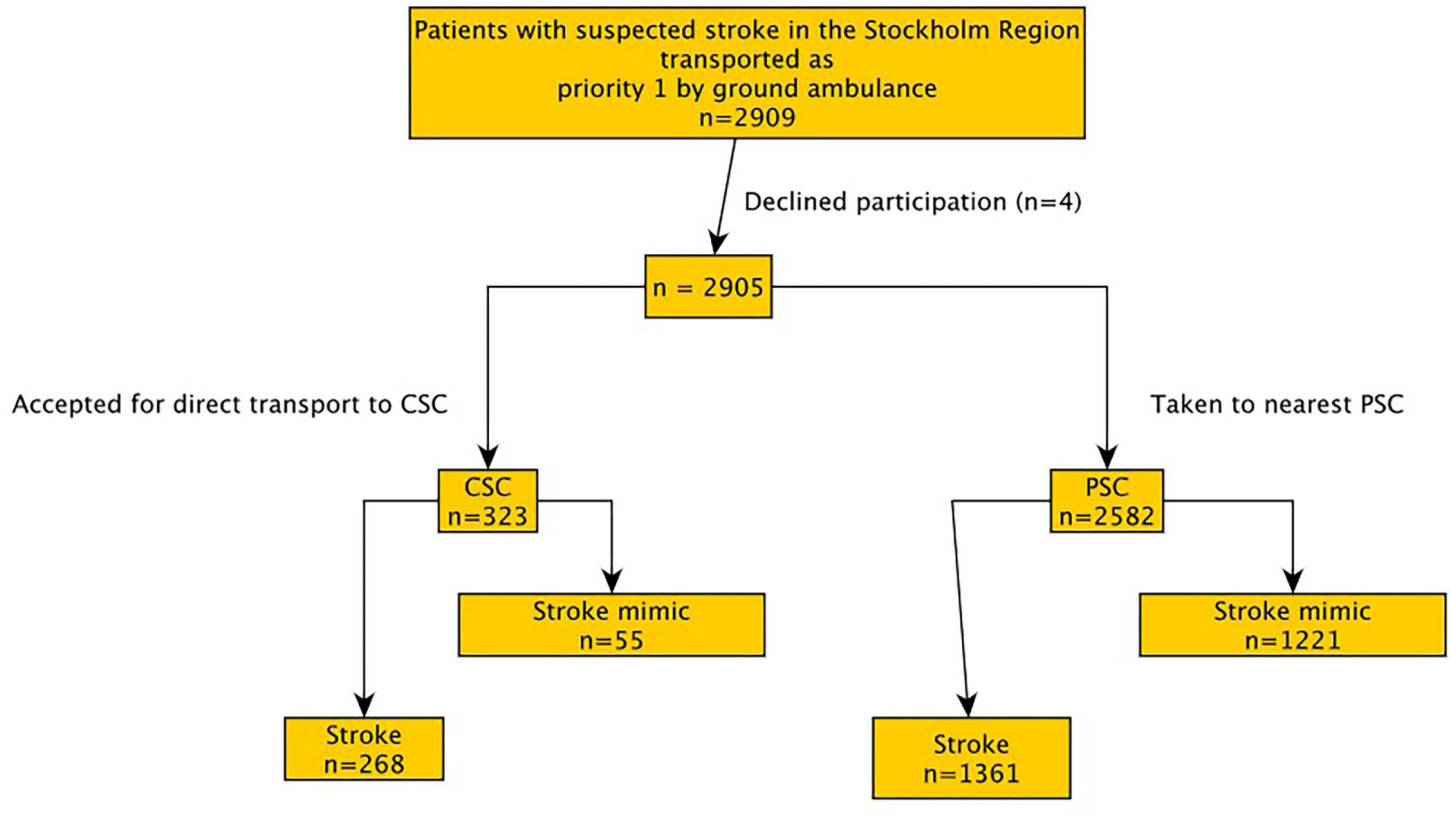
Patient distribution per study group.

Of 2,909 eligible patients, four declined to participate. Out of the remaining 2,905 patients, 1,276 (43.9%) received a stroke mimic diagnosis. Of the 1,276 patients with stroke mimic, 55 (4.3%) were triage-positive and 1,221 (95.7%) were triage-negative (Figure 2). As reported previously, the proportion of mimics among triage-positive patients was 55/323 (17%) and among triage-negative cases was 1,221/2,582 (47.3%) (*P* < 0.001) (5).

Table 1 shows baseline characteristics comparing TP mimics and TP stroke. Patients with TP mimic were younger than those with TP stroke (median age 64 vs. 75 years). There was a difference in gender distribution, 63.6% women vs. 44.8% men in the TP mimics and TP stroke, respectively. Patients with TP mimics had lower NIHSS scores (median seven) than those with TP stroke (median 15). In the FAST test, the patients with TP mimic less frequently had findings in face (38.2 vs. 72.4%) and speech (40 vs 78.7%) items compared to those with TP stroke.

**Table 1:** Comparison of triage-positive stroke mimics vs. triage-positive stroke.

	**Triage positive stroke mimics** ***N*** = **55**		**Triage positive stroke** ***N*** = **268**		
	***N***	**% or median** **(IQR)**	**N**	**% or median** **(IQR)**	***P*-value**
Demographics					
Age	55/55	64 (45–74)	268	75 (66–82)	<0.001
Sex, women	35/55	63.6%	120/268	44.8%	0.011
Baseline characteristics					
NIHSS Score	54/55	7 (3–10)	266/268	15 (9–19)	<0.001
FAST test status					
Positive face	21/55	38.2%	194/268	72.4%	<0.001
Positive arm	55/55	100%	268/268	100%	1
Positive leg	55/55	100%	267/268	99.6%	1
Positive speech	22/55	40%	211/268	78.7%	<0.001
A2L2 status					
A2L2 positive	55/55	100%	268/268	100%	1
A2L2 negative	0/55	0%	0/268	0%	1
A2L2 N/A	0/55	0%	0/268	0%	1
Known onset	42/55	76.4%	183/268	68.3%	0.235
Woke up with symptoms	14/55	25.5%	85/268	31.7%	0.359
ODT, min	52/55	86 (58–243)	265/268	86 (58–246)	0.88
Stroke subtype					
Cerebral infarction	N/A		190/268	70.9%	
ICH	N/A		68/268	25.4%	
SDH	N/A		3/268	1.1%	
TIA	N/A		7/268	2.6%	

A2L2, Arm 2-Leg 2-test; FAST, Face-Arm-Speech-Test; ICH, intracerebral hemorrhage; IQR, interquartile range; NIHSS, National Institute of Health Stroke Scale; ODT, onset-to-first-hospital-door time; SDH, subdural hematoma; TIA, transient ischemic attack.

As for patients with TN (Table 2), those with TN mimic were younger than those with TN stroke (median 73 vs. 78 years) and had lower NIHSS scores (median 2 vs. 4). TN Mimics and TN stroke differed significantly on all the FAST items: face (24.7 vs. 42.4%), arm (31.7 vs. 50.3%), leg (27.8 vs. 41.9%), and speech (50 vs. 62.2%).

**Table 2:** Comparison of triage-negative stroke mimics vs. triage-negative stroke.

	**Triage negative stroke mimics** ***N*** = **1,221**		**Triage negative stroke** ***N*** = **1,361**		
	**N**	**% or median (IQR)**	**N**	**% or median (IQR)**	***P*-value**
Demographics					
Age	1,221/1,221	73 (59–83)	1,361	78 (70–85)	<0.001
Sex, female	621/1,221	50.9%	644/1,361	47.3%	0.072
Baseline characteristics					
NIHSS Score	935/1,221	2 (0–6)	1,264/1,361	4 (2–9)	<0.001
FAST test status					
Positive face	281/1,138	24.7%	552/1,302	42.4%	<0.001
Positive arm	361/1,138	31.7%	655/1,302	50.3%	<0.001
Positive leg	316/1,138	27.8%	546/1,302	41.9%	<0.001
Positive speech	569/1,138	50%	811/1,303	62.2%	<0.001
A2L2 status					
A2L2 positive	56/1,221	4.6%	151/1,361	11.1%	<0.001
A2L2 negative	950/1,221	77.8%	1,075/1,361	79%	0.47
A2L2 N/A	215/1,221	17.6%	135/1,361	9.9%	<0.001
Known onset	626/1,221	51.3%	780/1,361	57.3%	0.002
Woke up with symptoms	518/1,221	42.4%	514/1360	37.8%	0.017
ODT, min	1,188/1,221	206 (81–625)	1,328/1,361	139 (69–467)	<0.001
Stroke subtype					
Cerebral infarction	N/A		825/1,361	60.7%	
ICH	N/A		174/1,361	12.8%	
SAH	N/A		30/1,361	2.2%	
SDH	N/A		57/1,361	4.2%	
TIA	N/A		269/1,361	18.7%	

A2L2, Arm 2-Leg 2-test; FAST, Face-Arm-Speech-Test; ICH, intracerebral hemorrhage; IQR, interquartile range; NIHSS, National Institute of Health Stroke Scale; ODT, onset-to-first-hospital-door time; SAH, subarachnoid hemorrhage, SDH, subdural hematoma; TIA, transient ischemic attack.

All patients with TP were, by definition, A2L2-positive; however, this was also seen in 56 (4.6%) of the TN mimic and 151 (11.1%) of TN stroke cases. Additionally, in 215/1,221 (17.6%) of the TN-mimic and 135/1,361 (9.9%) of the TN stroke cases, the A2L2 test was not applicable, indicating that they had a witnessed seizure, were unconscious, or had paresis of all the four limbs.

As seen in Table 3, the overall distribution of mimic diagnoses differed between the TP and TN mimic cases. We found significant differences in the occurrence of functional or other psychiatric disorders (more common in the TP group at 32.7 vs. 6.7%) and systemic infection (less common in the TP group, 1.8 vs. 13.1%) and a trend toward significance in syncope, hypotension, or other circulatory disorders (less common in the TP group, 1.8 vs. 9.7%).

**Table 3:** Comparison of diagnostic category distribution between patients with triage-positive mimic and those with triage-negative stroke mimic.

**Diagnostic category**	**Triage positive stroke mimics *N* = 55 (4.3%)**	**Triage negative stroke mimics *N* = 1,221 (95.7%)**		**All stroke mimics *N* = 1,276**
	***N* (%)**	***N* (%)**	***P*-value**	***N* (%)**
FND or other psychiatric diagnosis	18 (32.7%)	82 (6.7%)	<0.001	100 (7.8%)
Epileptic seizure	15 (27.3%)	264 (21.6%)	0.319	279 (21.9%)
Peripheral neuropathy	4 (7.3%)	75 (6.1%)	0.772	79 (6.2%)
Brain tumor	4 (7.3%)	50 (4.1%)	0.287	54 (4.2%)
Sequelae of previous stroke or other previous brain injury	3 (5.5%)	52 (4.3%)	0.511	55 (4.3%)
Primary headache disorder	2 (3.6%)	66 (5.4%)	0.765	68 (5.3%)
Other neurological diagnosis	2 (3.6%)	43 (3.5%)	1	45 (3.5%)
Musculoskeletal	2 (3.6%)	39 (3.2%)	0.696	41 (3.2%)
Systemic infection	1 (1.8%)	160 (13.1%)	0.011	161 (12.6%)
Syncope, hypotension, or other cardiovascular diagnosis	1(1.8%)	118 (9.7%)	0.055	119 (9.3%)
Drugs and alcohol	1 (1.8%)	82 (6.7%)	0.256	83 (6.5%)
Metabolic/endocrinological	1 (1.8%)	69 (5.7%)	0.361	70 (5.5%)
Peripheral vertigo	1 (1.8%)	47 (3.8%)	0.719	48 (3.8%)
Dementia	0	34 (2.8%)	0.397	34 (2.7%)
Head trauma	0	15 (1.2%)	1	15 (1.2%)
Other (miscellaneous) diagnosis	0	11 (0.9%)	1	11 (0.9%)
Transient global amnesia	0	8 (0.7%)	1	8 (0.6%)
Meningitis/encephalitis	0	6 (0.5%)	1	6 (0.5%)

FND, functional neurological disorder.

## Discussion

This observational study aimed to evaluate differences between triage-positive stroke mimics and stroke and triage-negative stroke mimics and stroke. We also aimed to establish whether the occurrence of specific stroke-mimicking conditions varies between triage-positive and triage-negative mimic groups.

An important finding was that the patients with stroke mimic were younger and had less severe symptoms, regardless of the triage status. While this pattern is well-established for mimics vs. true stroke (9), we were somewhat surprised that patients with TP stroke mimics and patients with TP stroke would have such a large difference in median NIHSS at 7 vs. 15. However, cases with occlusions of the M1 MCA segment or intracranial carotid artery in many studies have a median NIHSS of around 15–17 (10). The positive A2L2 test, being one of the requirements for TP status, would in itself generate an NIHSS subtotal of 4–8 points. It is clear from our findings that most patients with TP mimic had only these motor symptoms in isolation or with relatively minor additional symptoms. In terms of differences in distribution of stroke mimic diagnoses, functional neurological symptoms were common in TP mimics, while infections and syncope or hypotension were the more common causes of stroke mimics in TN mimics. The finding that functional neurological disorders (FNDs) were the most common diagnosis in the TP mimic group could be explained by the fact that patients with functional neurological symptoms often present with acute severe hemiparesis (11, 12), which is what the A2L2 test screens for. Moreover, our results indicate that the functional hemiparesis is a largely isolated symptom. Meanwhile, it is apparently difficult for ambulance staff to differentiate between FND and stroke even with the help of the mandatory teleconsultation with a stroke neurologist. In fact, FNDs are difficult to diagnose not only for ambulance staff but for physicians as well, making functional paresis the most common stroke mimic type to be inadvertently treated with IVT (13).

Our results are encouraging insofar that the 55 triage-positive stroke mimic cases directed to the CSC (i e, on average one per week) are unlikely to have caused serious bed capacity issues. Conversely, a large majority of mimics were correctly triaged to the nearest hospital, usually a PSC. One could consider whether our results could inform the teleconsultation process. We suggest that the finding of isolated hemiparesis commonly found in the FND group is insufficient to warrant a decline for direct CSC transport in such patients. This assessment is reinforced by a recent analysis by our group, which conducted a decision curve analysis to evaluate whether the triage algorithm could be improved using combinations of variables routinely available in the prehospital setting during the study period. This showed that classifying patients with isolated hemiparesis as triage-negative would not improve the precision of the system for LAO stroke or EVT (14). Cases with TP mimics had the same onset to hospital door time as TP stroke at just 86 min. Cases with dramatic symptoms and a short time from onset to alert of emergency services might have been difficult to decline for CSC routing. Meanwhile, the onset-to-first-hospital-door time (ODT) was more than 1 h longer in TN mimics than in TN stroke. Possibly, with longer time for the patient and bystanders to witness and observe early prehospital evolution and possibly additional symptoms, it might have been easier for a teleconsulted physician to correctly suspect a mimic in TN mimic cases even with hemiparesis (*n* = 56) and route them to a PSC instead of a CSC.

Regarding the higher frequency of infectious and circulatory stroke mimics among TN vs. TP mimics, this finding appears to reflect our clinical experience, as such mimic categories appear to relatively seldom generate major focal neurological syndromes.

There is an interesting pattern pertaining to sex differences in our study. The finding that female sex was overrepresented among the TP mimic group when compared to the TP stroke group could have been expected, as this has been seen in studies on IVT-treated stroke mimics (13). It appears that TP mimic cases were hard to distinguish from stroke in the prehospital setting even with a neurological teleconsultation. Meanwhile, the TN mimic and TN stroke groups did not have a significant sex difference. Importantly, we have previously shown that the SSTS performs equally between women and men, despite mimics being more common, overall, among women (15).

An important limitation of the study is the low number of individuals in the TP mimic group. This gives a limited statistical power for comparisons of this group vs. others. The low number in the TP group is in line with the previously reported high LAO and EVT specificity of the triage system (5). Furthermore, the limited number of variables describing clinical characteristics is a limitation, since it increases the risk of confounding factors not being identified and considered. This was due to the fact that the clinical characteristic data were collected from routine prehospital documentation, which prioritizes high data completeness over granularity.

## Conclusions

In the SSTS, those with triage-positive and those with triage-negative stroke mimics were younger and had less severe symptoms than patients with stroke. All patients with TP mimics had a hemiparesis but overall exhibited less severe symptoms against true stroke but more severe symptoms than those with TN mimics. Over-triage of functional paresis to the CSC was relatively common. Meanwhile, a large majority of the cases with minor symptoms experienced by stroke mimics and were triaged correctly by the SSTS to the nearest stroke center.

## Data availability statement

The raw data supporting the conclusions of this article will be made available by the authors, without undue reservation.

## Ethics statement

The studies involving human participants were reviewed and approved by the Swedish Ethical Review Authority approval 2017/374. The Ethics Committee waived the requirement of written informed consent for participation.

## Author contributions

AB and MM collected the dataset. MM designed and supervised the study. MS performed all the data processing, statistical analysis, and wrote the first draft of the manuscript. All authors contributed to the article and approved the submitted version.

## Funding

MM and CS received funding from the Innovation Fund of the Region Stockholm.

## Conflict of interest

The authors declare that the research was conducted in the absence of any commercial or financial relationships that could be construed as a potential conflict of interest.

## Publisher's note

All claims expressed in this article are solely those of the authors and do not necessarily represent those of their affiliated organizations, or those of the publisher, the editors and the reviewers. Any product that may be evaluated in this article, or claim that may be made by its manufacturer, is not guaranteed or endorsed by the publisher.
